# Passion projects and disorienting dilemmas

**DOI:** 10.1007/s40037-018-0453-6

**Published:** 2018-09-14

**Authors:** Zac Feilchenfeld, Ayelet Kuper

**Affiliations:** 1 Department of Medicine, University of Toronto, Toronto, Ontario Canada; 2 Sunnybrook Health Sciences Centre, Toronto, Ontario Canada; 3 The Wilson Centre, Toronto, Ontario Canada

In this issue of *Perspectives on Medical Education*, Fletcher et al. [1] share their experiences developing a curriculum on organ and tissue donation in Canadian medical schools. The co-first authors are medical students who led the creation, development and dissemination of the curriculum. Their article has two parts. The authors first describe their efforts and the background to their project, laying bare the challenges and benefits of a student-led curriculum development project. To their credit, this curriculum has been adopted at several medical schools across Canada. In the second part of their article, the students-as-educators reflect on how, in their experience, ‘active involvement in curricular design is a powerful way of integrating [CanMEDS] competencies’ [1].

The teaching and assessment of content that does not fall within the realm of traditional bio-scientific medical knowledge was problematic even before the advent of modern assessment frameworks and remains so today [2]. Contemporary assessment programs aim to drive learners to develop competency in, for example, collaboration, by placing collaboration within a workplace-based assessment framework, but the merits of this approach are still being evaluated. Some maintain that the non-medical expert or intrinsic CanMEDS roles (and similar constructs in other competency frameworks) are best learned ‘on-the-job’ through role modelling and workplace-based experience. In the Netherlands, Renting et al. showed that while the ‘professional attitudes and behaviours advocated in the CanMEDS framework were ubiquitous in clinical practice’, the CanMEDS roles, as named, ‘did not provide members of the community with a shared language in which to discuss resident performance, which may make the roles less useful than expected [3].’ Lurie et al.’s landmark systematic review demonstrated the challenges of reliable and valid measurement of the ‘other five competencies’ in the American ACGME framework (which may portend similar challenges for ‘the other six competencies’ in CanMEDS), while underscoring the potential benefits of curricular attention to these areas ‘previously perceived to be lacking in many training programs [4].’ In Canada, early work also sowed concern about the ability of teachers to teach and assess (and of learners to understand and appreciate) CanMEDS competencies [5, 6].

There are some non-medical expert competencies for which curriculum development has proven particularly challenging. Dharamsi et al. highlighted the anxiety and disappointment of educators who may have rejoiced in the increased attention, as per Lurie et al., to topics around social responsibility: ‘It has been over a decade since the formal adoption of competencies around health advocacy. Yet, how best to integrate them into medical education remains unclear, and little is known about how to effectively teach and assess learning in this area [7].’ As there continues to be significant interest in the teaching and learning of content relevant to developing certain non-medical expert CanMEDS competencies (in Canada and everywhere else CanMEDS and similar frameworks are being implemented), we would like to emphasize the potential contained in the experiences of Fletcher et al. [1]. In their paper, the authors candidly reflect on an evidently meaningful educational experience, cataloguing the development—to varying degrees—of the Advocate, Scholar, Collaborator, Professional, and Leader Roles. These reflections underscore the *transformative* potential of learning by doing non-traditional, non-clinical things as part of medical training—here, developing a curriculum. Transformative learning, as theorized by Mezirow and others [8], involves critical reflection through which one does not merely learn new skills, tasks, or knowledge, but experiences a change in perspective. This change requires ‘activities that stimulate self-reflection and engaged discourse, an internalization of humanistic values, a critical exploration of one’s own and society’s assumptions, biases, and values, and a commitment to enact the values that the profession espouses [9].’

In the transformative learning paradigm, learning is triggered when the learner is faced with a ‘disorienting dilemma’ (an experience that does not fit within a pre-existing meaning structure) that provokes critical self-reflection followed by additional theorized steps through which assumptions are questioned and perspective is transformed [8]. The teacher’s role in facilitating transformative learning is to ‘help learners become aware and critical of their own and others’ assumptions [10].’ Prompted by the dilemmas involved in developing, maintaining, and disseminating a new curriculum, the reflections of Fletcher et al. [1] represent enormous promise. The student-authors’ reflections are raw—they were not guided through a systemic critical reflective process (*cf. *Mezirow)—and yet it is clear that their experience was transformative.

That is certainly not to say that all students should participate in a curriculum development project in order to develop non-medical expert competencies, nor that these competencies *ought not* be learnt in the clinical environment. However, for the sometimes under-valued, or more ‘difficult-to-teach’, competencies like Advocacy, educators can be encouraged by the authors’ reflections. What cannot be known is the extent to which these students’ intrinsic motivation to pursue this project influenced their learning. Would mandatory projects—which will undoubtedly be viewed by many learners as extra ‘make-work’—provide the same educational benefits? How important was it that this was a passion project? Did their excitement and drive create the opportunity for an adequately ‘disorienting dilemma’, or would similar lessons be inevitable for any learner completing a comparable project outside the usual scope of medical training?

Perhaps the lesson here is that we should make space for students and residents to engage in passion projects, allowing enthusiasm, excitement, or serendipity to lead, through extra-curricular activities, to disorienting experiences that may cause students to question pre-existing assumptions or perspectives. In our contemporary medical training system, we want clinicians to do a variety of things, to be broadly competent in several domains, but we have learners ‘do’ a very narrow set of things, practically from start to finish in clinical training. We ask whether medical schools can make time in their curricula to enable learners’ passion projects, allowing them to do something a bit different. It would certainly be worth it if it turns out they would benefit as much as future Doctors Fletcher and Chen.
